# The ethylene response factor ERF1A regulates UV-C-induced delayed ripening in peach fruit

**DOI:** 10.1093/plphys/kiaf409

**Published:** 2025-09-22

**Authors:** Elpida Nasiopoulou, Michail Michailidis, Christina Skodra, Ioannis-Dimosthenis S Adamakis, Martina Samiotaki, Georgia Tanou, Christos Bazakos, Athanasios Dalakouras, Athanassios Molassiotis

**Affiliations:** Laboratory of Pomology, Department of Horticulture, Aristotle University of Thessaloniki, Thessaloniki-Thermi 57001, Greece; Laboratory of Pomology, Department of Horticulture, Aristotle University of Thessaloniki, Thessaloniki-Thermi 57001, Greece; Laboratory of Pomology, Department of Horticulture, Aristotle University of Thessaloniki, Thessaloniki-Thermi 57001, Greece; Department of Biology, National and Kapodistrian University of Athens, Athens 15784, Greece; Biomedical Sciences Research Center “Alexander Fleming”, Institute for Bioinnovation, Vari 16672, Greece; Institute of Soil and Water Resources, ELGO-DIMITRA, Thessaloniki-Thermi 57001, Greece; Joint Laboratory of Horticulture, ELGO-DIMITRA, Thessaloniki-Thermi 57001, Greece; Joint Laboratory of Horticulture, ELGO-DIMITRA, Thessaloniki-Thermi 57001, Greece; Institute of Plant Breeding and Genetic Resources, ELGO-DIMITRA, Thessaloniki-Thermi 57001, Greece; Department of Comparative Development and Genetics, Max Planck Institute for Plant Breeding Research, Cologne 50829, Germany; ELGO-DIMITRA, Institute of Industrial and Forage Crops, Larissa 41335, Greece; Laboratory of Pomology, Department of Horticulture, Aristotle University of Thessaloniki, Thessaloniki-Thermi 57001, Greece

## Abstract

Ultraviolet-C (UV-C) irradiation delays fruit ripening, yet the underlying mechanisms remain unclear. We investigated tissue-specific responses of peach fruit (*Prunus persica* L. Batsch) to UV-C by analyzing the peel and flesh separately. UV-C treatment altered central metabolism, promoted anthocyanin accumulation and coloration, and delayed ripening, as evidenced by reduced fruit softening and water loss. However, UV-C enhanced ethylene production and upregulated ethylene-related genes, indicating a reconfiguration of the ethylene response. Among UV-C-responsive genes, the APETALA2/Ethylene Response Factor (AP2/ERF) transcription factor family was most affected, with *Ethylene Response Factor 1A* (*ERF1A)* showing the strongest induction in the treated peel, suggesting its role as a key integrator of the UV-C-induced ripening delay. UV-C increased the levels of DNA 5-methylcytosine and RNA N6-methyladenosine in the peel, without altering cytosine methylation or causing mutations in ERF1A. Silencing ERF1A via RNA interference confirmed that it regulates ethylene production, softening, and ripening-associated metabolites. Immunolocalization revealed changes in the cell wall components of ERF1A-silenced fruit, including arabinogalactan, pectin, and xyloglucan. ERF1A-silenced peels exhibited elevated auxin and salicylic acid levels and reduced abscisic acid content. Additionally, ERF1A suppression altered the biosynthesis of sugars, phenolic compounds, and volatiles. We found extensive proteome reprogramming in ERF1A-silenced peels and identified putative ERF1A target genes that either contain ERF1A-binding sites or are associated with firmness, ethylene signaling, phytohormone metabolism, and color. Notably, *Carboxylesterase 11* (*PpCXE11*), *Carboxylesterase 13* (*PpCXE13*), and *Salicylic acid-binding protein 2* (*PpSABP2*) emerged as potential ERF1A targets. These findings identify ERF1A as a central regulator mediating UV-C-induced ripening delay through modulation of ethylene signaling and downstream ripening pathways.

## Teaser

UV-C irradiation delays peach fruit ripening through ERF1A-mediated reprogramming of ethylene signaling, hormone balance, and metabolic pathways.

## Introduction

Fleshy fruit ripening is a complex process regulated by external cues and internal signals, notably phytohormonal balance (Wang et al. 2018a). In climacteric fruits, ripening follows a classic linear model driven by the spatiotemporal expression of ethylene biosynthesis and signaling genes (Zhou et al. 2020). Accordingly, techniques manipulating ethylene biosynthesis, perception, or signal transduction are important strategies for fruit quality modulation (Severo et al. 2015). Functional characterization of genes encoding ripening-related transcriptional regulators, such as *RIPENING-INHIBITOR* (*RIN*), *MADS-box S1* (*MADS1*), *APETALA2a* (*AP2a*), *Ethylene Response Factor* 6 (*ERF6*), and *B3* (*ERFB3*), indicates that transcription factors (TFs) play key roles in transmitting ripening signals and regulating ethylene biosynthesis and signaling (Liu et al. 2015). Moreover, epigenetic modifications, such as DNA and RNA methylation, are also highly involved in fruit ripening (Ji and Wang 2023). Importantly, both TF activity and epigenetic modifications mostly regulate fruit ripening in an ethylene-dependent manner (Zhou et al. 2020), further emphasizing the importance of ethylene in climacteric ripening.

Ethylene Response Factors (ERFs) belong to the APETALA2 (AP2)/ERF superfamily and are considered direct regulators of ethylene-responsive gene expression (Zhou et al. 2020). Some ERFs function as activators of GCC-box-dependent transcription, while others serve as active repressors of basal transcription levels of reporter genes but also transactivate other TFs (Severo et al. 2015). Many ERFs regulate ethylene signaling and fruit ripening by directly binding to the GCC-box (with the core sequence AGCCGCC) or the dehydration-responsive element motif in the promoters of downstream target genes, such as 1-aminocyclopropane-1-carboxylate synthase (ACS) and 1-aminocyclopropane-1-carboxylate oxidase (ACO3) in model fruit species like tomato (*Solanum lycopersicum* L.) and apple (*Malus domestica* Borkh.) (Zhang et al. 2009; Li et al. 2016). In peach fruit (*Prunus persica* L. Batsch), several ERFs, including ERF1, may also function as either activators or repressors of ethylene biosynthesis genes, such as ACS and ACO, influencing their expression (Zhou et al. 2020). Furthermore, a link was observed between *ERF1A* expression and carotenoid accumulation in apricot fruit during ripening (Zhang et al. 2019), whereas ERF1 identified in plum fruit (*Prunus domestica*) appeared to be ethylene inducible and exhibited a ripening-related expression pattern (El-Sharkawy et al. 2009). Although these studies highlight that certain ERF members, including ERF1, are involved in fruit ripening across various *Prunus* species, it remains largely unknown whether different ERF family members play specific roles in mediating ethylene-dependent ripening and if they are regulated by external signals.

Ultraviolet-C (UV-C) irradiation is an important environmental signal that can affect fruit ripening (Guerrero-Beltrán et al. 2004). However, knowledge of the molecular shifts in response to UV-C stimuli during postharvest fruit ripening is still limited (Zhao et al. 2017), despite its substantial impact on ethylene signaling. As a stressor, UV-C can accelerate ethylene production and, consequently, induce ERF gene expression (Severo et al. 2015), such as *ERF1* upregulation in banana (*Musa nana* Laur.; Chen et al. 2022). It has been hypothesized that the delay in tomato ripening induced by UV-C treatment may result from the activation of ERFs, which may function as regulators of key metabolic pathways involved in the ripening process (Severo et al. 2015). Notably, most studies on the effects of UV-C treatment during ripening have focused primarily on the fruit flesh, likely due to the ability of UV-C to inhibit the activity of cell wall-degrading enzymes associated with softening (Kan et al. 2025). However, this emphasis overlooks the outer peel, a self-regulating protective barrier tissue with sensory and signaling capabilities that remains metabolically active throughout peach fruit ripening (Karagiannis et al. 2016). Previous studies have shown that UV-C treatment in peach peel triggers events associated with the initiation of ripening (Zhao et al. 2017), thereby highlighting the important role of the peel, alongside the flesh, in regulating the UV-C-dependent ripening process.

This work aims to enrich the current knowledge regarding the impact of UV-C in fruit ripening biology. To address this, we conducted a comprehensive analysis to elucidate physiological and transcriptomic events in response to postharvest UV-C application in peach, a climacteric fruit with a short storage capability due to its fast ripening or softening process (Tanou et al. 2017). Given the lack of tissue-specific data on UV-C ripening responses, our study investigates exocarp (peel) and mesocarp (flesh) tissues separately to uncover potential differences and shifts in the local and systemic dynamics of UV-C-induced ripening reprogramming between these 2 tissues. We report that UV-C can delay fruit ripening processes in concert with previously known ripening-related TFs, notably ERF1A. We found that silencing *Pp*ERF1A via RNAi in peach fruit led to significant alterations in several ripening parameters, particularly affecting hormone signaling and the volatile profile associated with the fruit's transition to ripening. This work provides a useful insight into the mechanisms of UV-C function not yet deciphered in fruit ripening biology.

## Results

### UV-C exposure delays the transition to ripening in peach fruit

**Figure 1.**
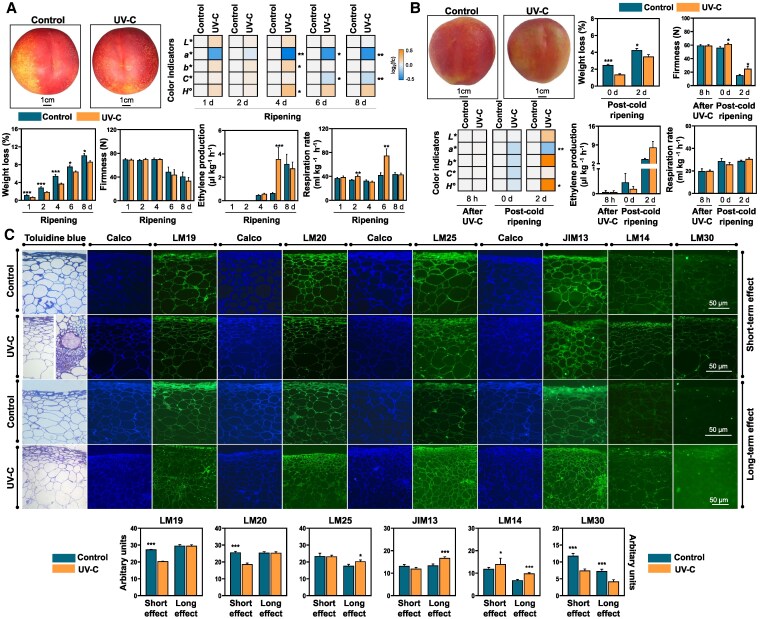
Phenotype and physiological data in peach fruit after UV-C treatment. Color indicators (L*, a*, b*, C*, and *H*°), weight loss (18 fruits), flesh firmness, ethylene production, and respiration rate (3 fruits × 3 replicates) **A)** during ripening at 20 °C for 1, 2, 4, 6, and 8 d after UV-C treatment as well as **B)** after 8 h of UV-C application or after 0 and 2 d of post-cold ripening at 20 °C in “Luciana” fruit. Heatmaps demonstrating the ratio of color indicators between UV-C and control transformed into log_2_ and depicted with color scale (from blue to orange). **C)** Microscopy images, fluorescent immunolocalization, and estimation of fluorescence intensity in stained/immunostained fruit sections (5 sections of 3 fruits) after 8 h (short-term effect) and after 3 d at 20 °C (long-term effect) following UV-C treatment. The vertical lines in bar graphs represent the standard error of the mean. For all graphs, the asterisk symbol (*) indicates significant differences between treatments according to Student's *t*-test (*P ≤ 0.05, **P ≤ 0.01, ***P ≤ 0.001). Fc, fold change; Calco, calcofluor-white.

To characterize the ripening process, several parameters were assessed during fruit exposure to room temperature following UV-C application in the absence or presence of cold treatment. In UV-C-exposed fruit, the peel color parameter a* decreased at 4, 6, and 8 d of ripening, as well as at 2 d of post-cold ripening (Fig. 1A; Fig. 1B). Similarly, C* declined at 6 and 8 d (Fig. 1A), while *H°* increased at 4 d of ripening and at 2 d following cold storage (Fig. 1A; Fig. 1B). The most prominent effect of UV-C was the reduced water loss of treated fruit at all ripening stages and conditions examined (Fig. 1A; Fig. 1B). UV-C-treated flesh firmness remained at higher levels during post-cold ripening (Fig. 1B). Εthylene production rate was 5 times higher in UV-C-treated fruit at 6 d, while respiration was elevated at both 2 and 6 d of ripening following UV-C (Fig. 1A). Ion leakage was increased in UV-C-exposed peels at 2 and 4 d of ripening, as well as at 0 and 2 d of post-cold ripening, indicating that a short UV-C exposure induces long-term stress conditions in peel tissues (Supplementary Fig. S1).

The reduction in fruit softening by UV-C (Fig. 1B) was linked to changes in arabinogalactan, pectin, and xyloglucan epitopes (Supplementary Table S1) through immunolocalization and fluorescent microscopy at 8 h of ripening (referred to as “short-term UV-C effect”), as well as 3 d after UV-C treatment (referred to as “long-term UV-C effect”) (Fig. 1C). As a long-term UV-C effect, hypodermal cells of treated fruit were increased in number but reduced in size (Fig. 1C). The use of 2 monoclonal antibodies directed to differing methylesterification states of pectic homogalacturonan (HG) indicated that both unmethylesterified HG epitope (LM19) and highly methylesterified HGs detected by LM20 antibody were generally reduced 8 h after UV-C (Fig. 1C). Localization of arabinogalactan protein (AGP) epitopes reveals that JIM13 signal was more abundant in UV-C-exposed fruit at 3 d of ripening (Fig. 1C). UV-C-treated fruit were highly labeled with LM14 regardless of the ripening time. Signals of LM30 decreased after both short and long UV-C periods. In response to the long-term UV-C effect, the fluorescence of the xyloglucan-specific antibody LM25 was highly abundant (Fig. 1C).

### Transcript profiling reveals aspects of the tissue-specific function of UV-C

**Figure 2.**
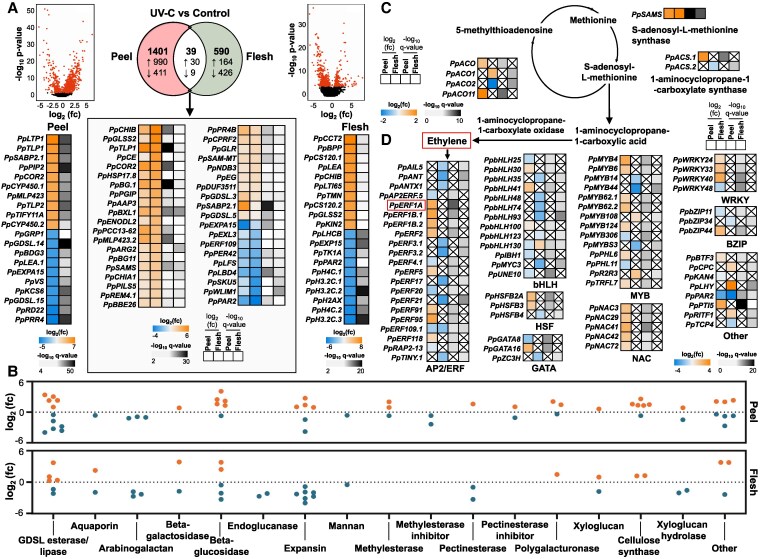
DEGs between control and UV-C-treated exocarp (peel) and mesocarp (flesh) tissues of the fruit 8 h after UV-C treatment. **A)** Heatmap of the top 10 up- and downregulated genes in both tissues. Volcano plot: Each point in the plot represents a gene. The red points represent the DEGs, while the black ones represent the unchanged genes. Venn diagram showing the overlap of DEGs between peel and flesh; upward arrows indicate upregulated genes and downward arrows indicate downregulated genes. Heatmap (inside the box) of commonly expressed DEGs (39 DEGs) in both tissues. **B)** Differentially expressed cell-wall genes categorized into groups. The orange points depict the upregulated genes, while the blue points depict the downregulated genes. Heatmap of **C)** DEGs involved in ethylene biosynthesis and of **D)** TFs that differentially expressed in UV-C-treated peach tissues. Heatmap column explanation (from the left to the right); log_2_ (fold change) and −log_10_ (*q* value) between UV-C and control peach fruit in peel and flesh samples. The increase is indicated with orange, while the decrease is denoted with blue (see color scale). Gene abbreviations are described in **Supplementary Table S3**. Fc, fold change.

RNA-seq analysis was conducted to examine the effect of UV-C on the peel and flesh tissues 8 h after application (Supplementary Table S2). The transcriptomic impact of UV-C in the peel was stronger than in the flesh tissue, as shown by the 1,440 differentially expressed genes (DEGs) identified in peel (1,020 up- and 420 downregulated) compared to 629 DEGs found in the flesh (194 up- and 435 downregulated) (Fig. 2A). The present data showed that gene induction is a general response of the peel to UV-C exposure, since an extensive upregulation of several DEGs was detected. In contrast, the UV-C-treated flesh displayed a general downregulatory trend for several DEGs, indicating the existence of a tissue-dependent regulation of UV-C (Fig. 2A). The top 10 up- and downregulated DEGs (control vs UV-C) in the peel and flesh separately are shown (Fig. 2A). Distinct genes, such as cytochrome P450 (*PpCYP450.1* and *PpCYP450.2*) and choline-phosphate cytidylyltransferase 2 (*PpCCT2*) were strongly upregulated in the peel and flesh tissues, respectively, due to UV-C, while the expression of genes encoding GDSL esterase/lipase (peel tissue; *PpGDSL.14* and *PpGDSL.15*) and expansin B15 (flesh tissue; *PpEXP15*) was decreased (Fig. 2A). Markedly, 39 DEGs (30 and 9 genes up/downregulated, respectively), including desiccation-related protein PCC13-62 (*PpPCC13-62*) and ethylene-responsive TF ERF109 (*PpERF109*), were affected by UV-C in both tissues (Fig. 2A) and could be considered hallmarks of UV-C signaling. RNA-seq expression dynamics were validated by RT-qPCR (*R*^2^ = 0.9263; Supplementary Fig. S2).

Transcriptomic analysis revealed numerous cell wall-related genes affected by UV-C that belong to several categories, including arabinogalactans, expansins, pectinesterases, etc. (Figure 2B). As an example, *PpAGP1.2*, *PpAGP18*, and *PpAGP31* were downregulated in the flesh, while *PpAGP1.1*, *PpAGP16.1*, and *PpAGP16.2* were downregulated in the UV-C-treated peel (Fig. 2B; Supplementary Table S3). Besides, UV-C regulated several genes in both tissues that participate in ethylene biosynthesis, such as *PpACS.1*, *PpACS.2*, *PpACO*, *PpACO1*, *PpACO2*, *PpACO11*, and S-adenosylmethionine synthase (*PpSAMS*), the latter showing >1.5 log_2_(fold change) upregulation in both tissues (Fig. 2C). Additionally, we analyzed the impact of UV-C on the expression of all DEGs annotated as putative TFs (Fig. 2D). Several UV-C-responsive TFs were identified, most of them belong to AP2/ERF, bHLH, HSF, GATA, MYB, NAC, WRKY, and bZIP families (Fig. 2D).

### Changes of primary and secondary metabolites elicited by UV-C

**Figure 3.**
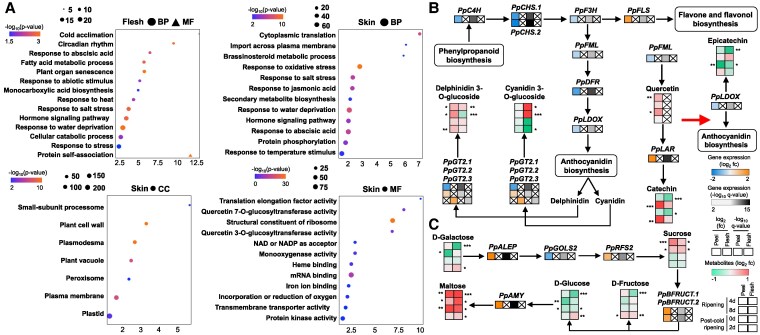
GO enrichment analysis and metabolic pathways in response to UV-C. **A)** GO enrichment analysis for upregulated genes in peel and flesh of peach fruit. The size of the spots represents the number of DEGs in each category, while in flesh, the circle depicts the domain of Biological Process and the triangle the domain of Molecular Function. The color scale (from blue to orange) depicts −log_10_  *P*-value. Changes in **B)** flavonoid and anthocyanin biosynthesis as well as **C)** galactose metabolism in response to UV-C. Gene expression is according to fold change (log_2_) and −log_10_ (*q* value). The increase is indicated with orange, while the decrease is with blue (see color scale). Heatmap column explanation (from the left to the right); log_2_ (fold change) and −log_10_ (*q* value) between UV-C and control peach fruit, in exocarp and mesocarp samples. Metabolite accumulation, represented as fold change (log_2_), is illustrated using a color scale ranging from green to red for peel and flesh samples at 4 and 8 d of ripening, as well as at 0 and 2 d of post-cold ripening. BP, biological process; MF, molecular function; CC, cellular component; Fc, fold change.

To define the biological process, molecular function, and cellular component associated with UV-C, we performed a Gene Ontology (GO) enrichment analysis of all DEGs in the peel and flesh separately. The analysis revealed an enrichment in the “Secondary metabolite biosynthesis” bin among the identified DEGs (Fig. 3A). Metabolomic analysis disclosed several tissue-metabolic changes after UV-C treatment (Supplementary Fig. S3; Supplementary Table S4; Supplementary Table S5). For instance, both catechin and procyanidin B1 levels were lower in the flesh but higher in the peel tissue of UV-C fruit (Supplementary Fig. S3B). Rutin and quercetin-3-*O*-glucoside were only detected in the peel, exhibiting a UV-C-inducible increase. Cyanidin levels were increased in the flesh during ripening after UV-C but decreased during post-cold ripening, while delphinidin was induced by UV-C in the peel during most ripening conditions (Supplementary Fig. S3B). Regarding central metabolism, oxoproline increased in the flesh and decreased in the peel during ripening, while its abundance was reduced in both tissues during post-cold ripening (Supplementary Fig. S3A).

Since the functional enrichment analysis highlighted the category of secondary metabolite biosynthesis (Fig. 3A) and a substantial alteration of phenolic compounds was observed in UV-C fruit (Supplementary Fig. S3), we focused on polyphenolic metabolism, including transcriptomic and metabolomic data (Fig. 3B). Several genes involved in flavone and flavonol biosynthesis, including trans-cinnamate 4-monooxygenase (*PpC4H*), chalcone synthase (*PpCHS.1* and *PpCHS.2*), flavanone 3-hydroxylase (*PpF3H*), and flavonol synthase (*PpFLS*) were affected in the peel by UV-C. Various UV-C-related polyphenolic pathways were affected either by the tissue or cold treatment. For example, leucoanthocyanidin reductase (*PpLAR*) expression and catechin accumulation were increased in the peel compared to the opposite trends found in the flesh. The accumulation of cyanidin and delphinidin with a coordinated increase in anthocyanidin 3-*O*-glucosyltransferase 2 (*PpGT2.1*, *PpGT2.2*, and *PpGT2.3*) expression in UV-C flesh during ripening, accounted for the enhanced cyanidin 3-*O*-glucoside accumulation; however, this metabolite route was suppressed by cold (Fig. 3B). The present data also showed that sugar regulation was altered by UV-C (Supplementary Fig. S3). UV-C led to a reduction in D-galactose during ripening, while it stimulated sucrose and maltose in both tissues. D-fructose was found at higher levels in both tissues during post-cold ripening (Fig. 3C; Supplementary Fig. S3A). Consistent with this, genes involved in the biosynthesis of sucrose (i.e. aldose 1-epimerase, *PpALEP* and galactinol-sucrose galactosyltransferase 2, *PpRFS2*), maltose (alpha amylase, *PpAMY*; >2 log_2_ (fc)), and D-fructose (i.e. beta-fructofuranosidases, *PpBFRUCT.1* and *PpBFRUCT.2*) were provoked by UV-C (Fig. 3C).

### UV-C induces global epigenetic and epitranscriptomic changes in peach fruit

**Figure 4.**
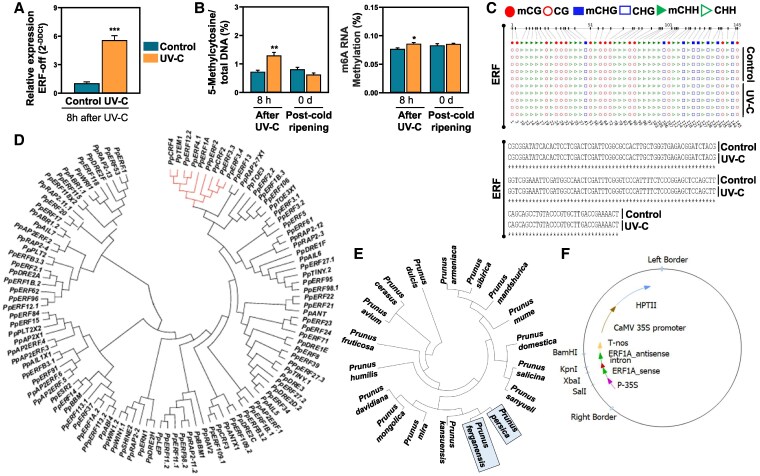
Characterization of ERF1A in terms of expression, methylation, mutation, and phylogenetic traits. **A)** Relative expression of ERF1A at 8 h after UV-C treatment (3 fruits × 3 replicates). **B)** Quantification of global DNA and m6A% RNA methylation at 8 h after UV-C application and at the initiation of post-cold ripening (0 d). For (A) and (B), the vertical lines represent the standard error of the mean, while the asterisk symbol (*) indicates significant differences between treatments according to Student's *t*-test (*P ≤ 0.05, **P ≤ 0.01, ***P ≤ 0.001). **C)** Sequencing and bisulfite sequencing of ERF1A at 1 d after UV-C treatment. **D)** Phylogenetic analysis of the *P. persica* members of AP2/ERF superfamily. The red lines indicate the members clustered with ERF1A. **E)** Phylogenetic analysis of ERF1A among different *Prunus* species. The light blue rectangles depict the species found closely to *P. persica*, based on the sequences of ERF1A homologs. Phylogenetic trees in graphs (D) and (E) are shown as cladograms, and branch lengths are not proportional to evolutionary distance. **F)** pCambia vector containing the cassette “ERF1A sense + intron + ERF1A antisense” under the control of the CaMV35S promoter.

Given that UV may lead to epigenetic modifications in plants (Willing et al. 2016; Xiang et al. 2017), we examined whether UV-C could induce epigenetic (DNA methylation) and epitranscriptomic (RNA methylation) modifications in the peel directly exposed to UV-C. By measuring levels of DNA 5-methylcytosine (5mC) and RNA N6-methyladenosine (m6A), we observed that both global DNA and RNA methylation were stimulated in the peel 8 h after UV-C (Fig. 4B), proposing that DNA 5mC and RNA m6A represent epigenetic markers of UV-C functions in plants.

### The expression patterns of ERF1A TF were considerably affected by UV-C

Because any transcriptomic reprogramming requires the intervention of TFs (Osorio et al. 2020), we performed a TF analysis. Strikingly, the family with the highest number of differentially expressed TFs was AP2/ERF, while ERF1A in this family displayed the greatest (−log_10_  *q*-value 12.7) upregulation levels in UV-C peel (>2.5 log_2_ (fc); Fig. 2D; Fig. 4A), highlighting it as a candidate gene that underpins the observed UV-C ripening signaling and response. Previous studies have established that ERF1 regulates the expression of ACS and ACO by directly binding to the GCC-box and contributes to ethylene and climacteric ripening signaling (Zhou et al. 2020). Therefore, we investigate the functional significance of ERF1A in UV-C ripening signaling.

### UV-C treatment did not induce epigenetic or genetic changes in ERF1A

Given (i) the observed UV-C-induced methylation in peel samples (Fig. 4B) and the reported sensitivity of ERF binding sites to methylation (Han et al. 2016; Mielko et al. 2023) and (ii) UV-C's potential to cause mutations that affect TF binding (Nakamura et al. 2021), along with evidence that ERF-related mutations can alter gene expression and stress responses (Sun et al. 2010), we examined whether UV-C induces methylation and/or mutation of ERF1A to clarify its functional role in UV-C-driven ripening. Bisulfite sequencing showed no cytosine methylation in ERF1A, and coding region sequencing revealed no UV-C-induced mutations (Fig. 4C).

### Phylogenetic analysis of *P. persica* ERF family and ERF1A across *Prunus* species

Phylogenetic analysis was performed to investigate the evolutionary relationship between *PpERF1A* and other members of the AP2/ERF family in *P. persica* (Fig. 4D; Supplementary Table S6). Based on their coding sequences (CDS), *PpERF1A* clustered with ethylene-responsive TF CRF4 (*PpCRF4*), AP2/ERF and B3 domain-containing transcription repressor TEM1 (*PpTEM1*), *PpERF12.2*, *PpERF4.1*, *PpERF2*, *PpCRF2*, *PpERF3.3*, and *PpERF3.4*. The evolutionary relationship of ERF1A across different *Prunus* species was also examined (Fig. 4E; Supplementary Table S6). This analysis showed that *P. persica* ERF1A (*PpERF1A*) grouped with its homolog from *P. ferganensis* and showed similarity to the ERF1A-like gene from *P. kansuensis*. Other closely related species based on ERF1A homologs included *P. mira*, *P. mongolica*, and *P. davidiana* (Fig. 4E).

### ERF1A silencing reveals its key role in peach fruit ripening

**Figure 5.**
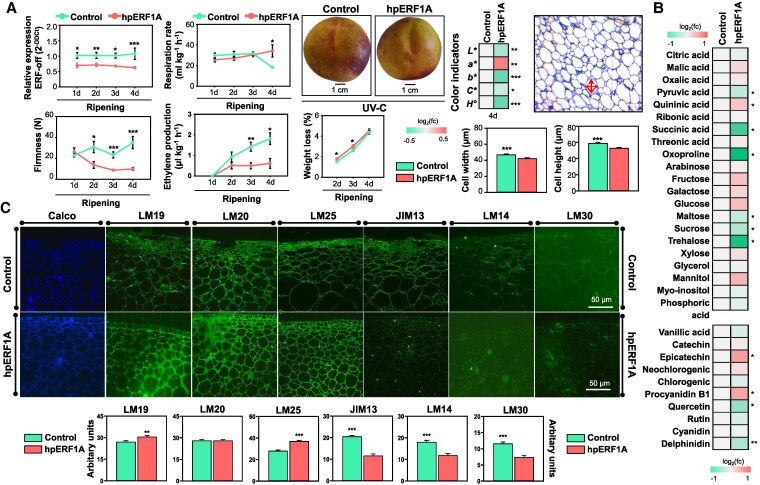
Changes in peach ripening features in response to ERF1A silencing prior to UV-C treatment. **A)** ERF1A relative expression, weight loss (18 fruits), color indicators (L*, a*, b*, C*, and *H*°), flesh firmness, respiration rate, and ethylene production (3 fruits × 3 replicates) in ERF1A-silenced “Morsiani 90” fruit across ripening after UV-C treatment. Fruit phenotypes (at 4 d of ripening) and heatmap demonstrating the ratio of color indicators between UV-C-treated control and ERF1A-silenced peach fruit transformed into log_2_ and depicted with a color scale (from green to red). Cell height and cell width of ERF1A-silenced peach fruit cells after UV-C treatment are indicated with red arrows. **B)** Heatmap demonstrating primary and secondary metabolite ratio between UV-C-treated control and ERF1A-silenced peach fruit at 4 d of ripening after UV-C treatment transformed into log_2_ and depicted with a color scale (from green to red). **C)** Microscopy images using calcofluor-white staining, LM19, LM20, LM25, JIM13, LM14, and LM30 antibody labeling of ERF1A-silenced peach fruit sections after UV-C treatment. Fluorescence intensity images and estimation of fluorescence intensity are also depicted. For all graphs, the asterisk symbol (*) indicates significant differences between treatments according to Student's *t*-test (*P ≤ 0.05, **P ≤ 0.01, ***P ≤ 0.001). Calco, calcofluor-white; Fc, fold change.

To characterize the potential role of ERF1A in UV-C-associated ripening, we sought to silence ERF1A expression in the peel. To do that, we performed an RNAi assay through vacuum agro-infiltration, which leads to transient silencing of the target gene (ERF1A; Fig. 4F). Thus, we performed the vacuum agroinfiltration RNAi assay 3 d before UV-C treatment in nectarine “Morsiani 90,” since interference is usually initiated 3 d post-agroinfiltration (Dalakouras et al. 2019). It is worth noting that UV-C treatment also postpones ripening in “Morsiani 90” fruit (Supplementary Fig. S4) and strongly upregulates ERF1A expression in their peel (almost 5 times higher; Supplementary Fig. S5). To evaluate the silencing efficiency of agroinfiltrated tissue, ERF1A expression levels were measured by RT-qPCR at 1, 2, 3, and 4 d of ripening after UV-C (4, 5, 6, and 7 d following ERF1A silencing, respectively) in the ERF1A-RNAi peel (hpERF1A) compared to the control (hpGFP). ERF1A downregulation was observed in infiltrated peel samples at all time points of the examined period, signifying successful ERF1A silencing (Fig. 5A).

We next examined how ERF1A silencing affects peach fruit ripening. ERF1A-silenced fruit had lower ethylene production at 3 and 4 d of ripening after UV-C but higher respiration activity at 4 d (Fig. 5A). As arose from the peach phenotype, the ERF1A silencing alters color development, particularly at 4 d of ripening following UV-C treatment (Supplementary Fig. S6). Indeed, L*, b*, C*, and *H°* were reduced, while indicator a* was increased (Fig. 5A). The analysis of fruit ripening also showed that ERF1A-RNAi fruit exhibited higher weight loss at 2 and 3 d of ripening (Fig. 5A). Since weight loss may be associated with changes in fruit cell size (Karim et al. 2022), histological analysis was performed to analyze the cell size characteristics of ERF1A-silenced tissue. We found that ERF1A-RNAi fruits exhibited lower cell height and cell width (Fig. 5A). In parallel, significant metabolic changes in response to ERF1A silencing were detected at 4 d of ripening after UV-C. The content of several sugars, such as maltose and sucrose, as well as organic acids, like pyruvic and succinic acid, were reduced in ERF1A-silenced peel (Fig. 5B). Silenced fruit also had increased epicatechin and procyanidin B1 levels, while the opposite was noticed for several polyphenols (e.g. quercetin 3-D-galactoside and delphinidin; Fig. 5B). Meanwhile, ERF1A-silenced fruit showed consistently lower flesh firmness, especially at 4 d of ripening, with hpERF1A fruit measuring ∼10 N versus ∼35 N in hpGFP controls (Fig. 5A). The immunofluorescent labeling of LM19 and LM25 epitopes was more intense in ERF1A-silenced fruit, following ERF1A suppression (Fig. 5C). Nonetheless, the detection levels of AGPs were lower in ERF1A fruits, since all AGP-related antibodies, namely LM14, LM30, and JIM13 had a reduced presence (Fig. 5C). Collectively, the ripening acceleration and the metabolic and anatomical changes detected in ERF1A-silenced fruit signify that ERF1A broadly impacts peach fruit ripening following UV-C treatment.

### ERF1A silencing altered protein shifts and partially reversed UV-C ripening outcome

**Figure 6.**
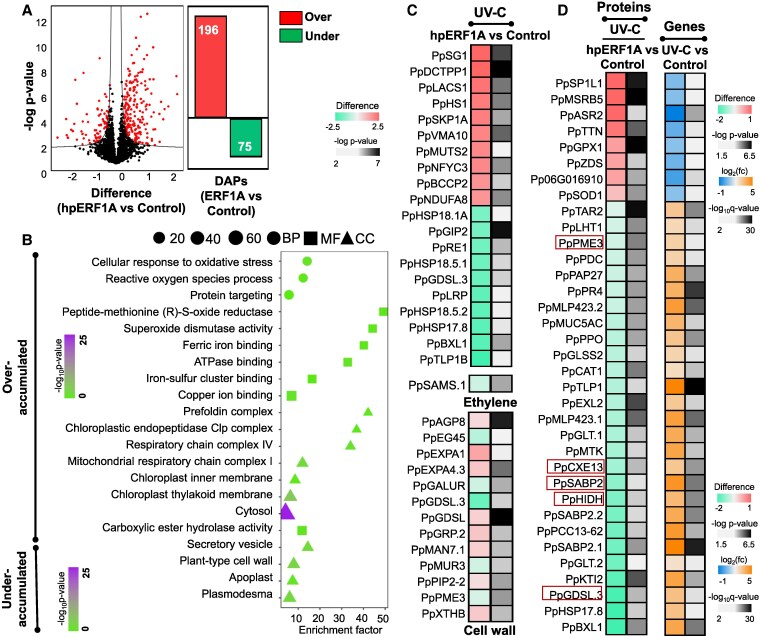
The impact of ERF1A silencing on the abundance of ripening-related proteins. DAPs of UV-C-treated fruit peel after ERF1A silencing. **A)** Volcano plot: each point in the plot represents a protein. The red points represent the DAPs, while the black ones represent the unchanged proteins. Bar plot shows the number of over- and underaccumulated proteins. **B)** GO enrichment analysis for over- and underaccumulated peel proteins. The size of the spots represents the number of DAPs in each category, while the shape represents the domain (circle—Biological Process, square—Molecular Function, and triangle—Cellular Component. The color scale (from green to red) depicts −log_10_  *P*-value. **C)** Heatmap of the top 10 up- and down-accumulated proteins along with the ethylene- and cell wall-related DAPs. **D)** Heatmap of DAPs between hpERF and hpGFP, and DEGs between UV-C and control treatment. Heatmap demonstrating the difference of DAPs between hpERF and hpGFP depicted with a color scale (from green to red), and the ratio of DEGs between UV-C and control transformed into log_2_ and depicted with a color scale (from blue to orange). Significant differences in DAPs are indicated with −log *P*-value, while differences in DEGs are indicated with −log_10_  *q*-value. BP, biological process; MF, molecular function; CC, cellular component; Fc, fold change.

Given that the proteome is closer to the fruit phenotype than the genome or the transcriptome (Chan 2013), we performed proteomic analysis in ERF1A-silenced peel at 1 and 4 d of ripening after UV-C (Fig. 6). The number of differentially accumulated proteins (DAPs) between ERF1A-silenced and control samples was 4 and 271 DAPs after 1 and 4 d, respectively (Supplementary Table S7). We postulated that the 4-d proteomic data, rather than the 1-d ones, reflect a strong proteomic effect of ERF1A silencing on ripening. Therefore, we focused our study on the 4-d samples to gain insight into the altered ripening processes of ERF1A-silenced fruit (Fig. 5). Intriguingly, the 4 DAPs detected at 1 d (10 kDa chaperonin mitochondrial PpCPN10, acyl-CoA-binding protein PpACBP, cationic peroxidase 1 PpCP1, and target of Myb protein 1 PpTOM1) were also found at the 4-d data (Supplementary Table S7), proposing that these proteins could represent targets of ERF1A. Among the identified DAPs (271 proteins), 196 proteins were overaccumulated and 75 were underaccumulated (Fig. 6A). The most enriched GO categories of the DAPs are provided in Fig. 6B, among which were chloroplast membrane for CC, copper ion and ferric ion binding for MF, and response to oxidative stress for BP. The abundance of the top 10 over- and underaccumulated proteins in ERF1A-silenced fruit is shown in Fig. 6C, including stress-response A/B barrel domain-containing protein (PpHS1) and heat shock proteins (PpHSP18.1A, PpHSP18.5.1, PpHSP18.5.2, and PpHSP17.8). Consistent with ethylene and softening changes (Fig. 5A), the abundance of PpSAMS.1 was decreased (Fig. 6C), while the accumulation of several cell wall proteins, such as 2 expansin proteins (PpEXPA1 and PpEXPA4.3), was increased by ERF1A silencing (Fig. 6C).

This work aims to enrich the knowledge regarding the interaction of UV-C and ERF1A during ripening. Thus, data from the proteomic experiment performed under UV-C (hpERF1A vs unsilenced control) were compared with the RNA-seq dataset concerning the peel (UV-C vs untreated control) (Fig. 6D). This approach, despite the different experimental conditions of each data set, revealed numerous UV-C-responsive genes whose corresponding proteins displayed different accumulation trends following ERF1A suppression. For example, the abundance of 8 proteins, including methionine sulfoxide reductase B5 (PpMSRB5), was increased in ERF1A fruit, while the corresponding genes were downregulated in UV-C-exposed ones. Also, 27 proteins, including carboxylesterase 13 (PpCXE13), salicylic acid-binding protein 2 (PpSABP2), and 2-hydroxyisoflavanone dehydratase (PpHIDH), were underaccumulated in ERF1A fruit, while their genes were upregulated by UV-C (Fig. 6D). These results indicate that ERF1A silencing before UV-C treatment, apart from altering ripening, may reverse a large part of UV-C signaling.

### Identification of the target candidate genes of ERF1A

**Figure 7.**
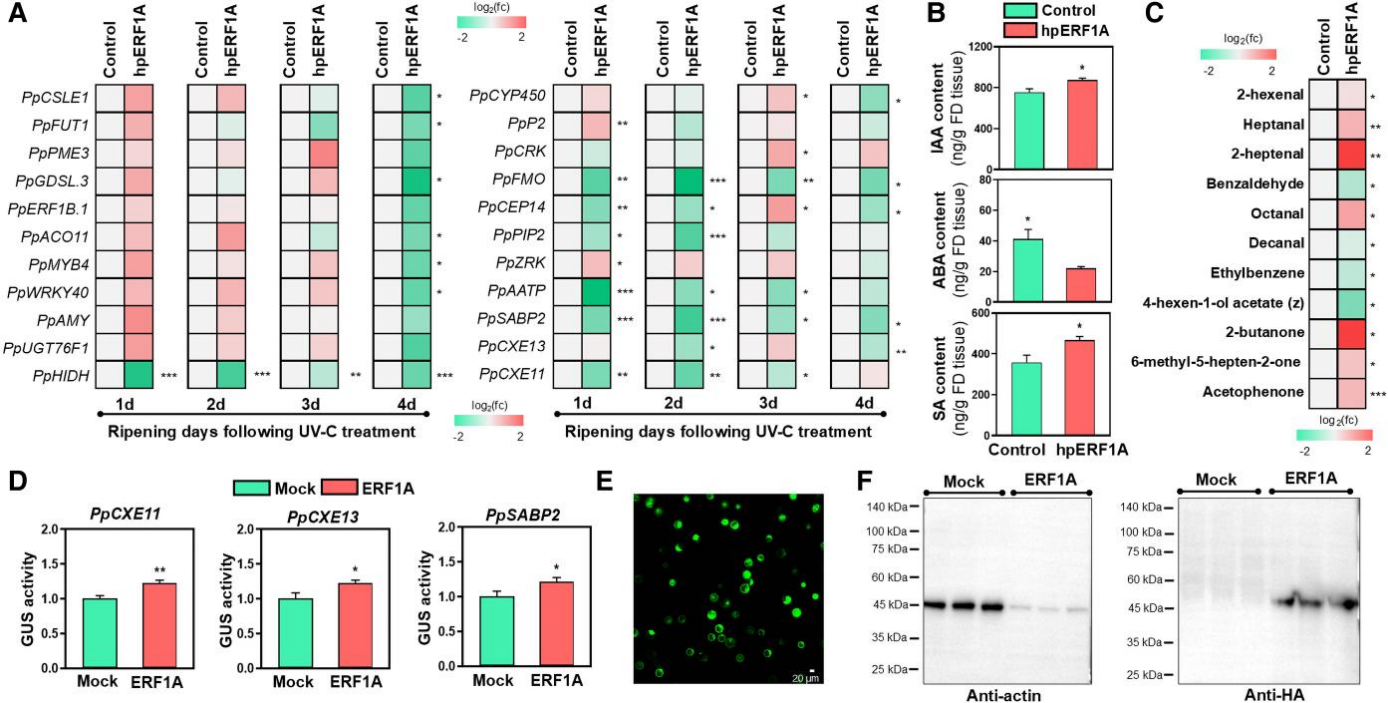
Identification of the target candidate genes of ERF1A. **A)** Expression analysis of putative target genes of ERF1A in peel samples following ERF1A silencing in peach fruit during ripening after UV-C treatment. **B)** Steady-state levels of IAA, ABA, and SA in ERF1A silencing peel tissue (3 fruits × 3 replicates) at 4 d of ripening following UV-C treatment. The ratio between control and ERF1A silencing samples in heatmaps is transformed into log_2_ and depicted with a color scale (from green to red). **C)** Changes in volatile organic compounds in ERF1A silencing peel at 4 d of ripening following UV-C treatment. **D)** Transactivation activity of ERF1A (3 measurements × 3 replicates) using GUS reporter assay on promoters of selected genes (PpCXE11, PpCXE13, and PpSABP2). Values are the average of normalized GUS activity relative to mock control. For graphs (A), (B), (C), and (D), the asterisk symbol (*) indicates significant differences between treatments according to Student's *t*-test (*P ≤ 0.05, **P ≤ 0.01, ***P ≤ 0.001). **E)** Fluorescent imaging expression in Αrabidopsis protoplast by transforming GFP-containing vector. **F)** Western blot analysis of ERF1A and mock control after incubating with anti-actin and anti-HA antibodies. Fc, fold change; FD, freeze-dried; M, marker.

**Figure 8.**
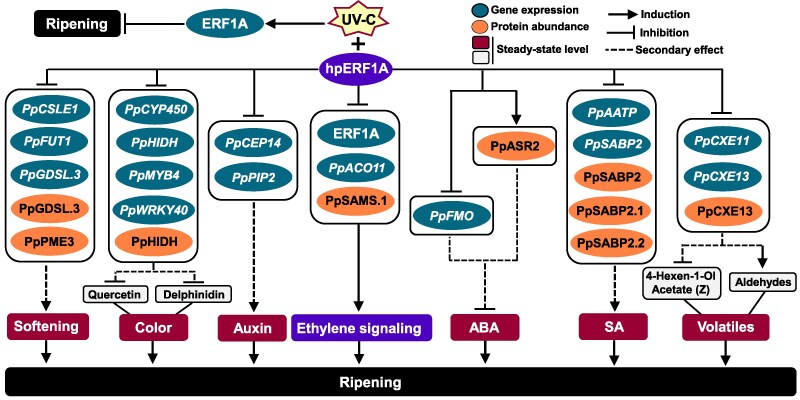
Proposed model of ERF1A-mediated UV-C response in peach fruit ripening. UV-C treatment activates ERF1A expression in the peel, triggering downstream metabolic changes that delay ripening. By contrast, silencing of ERF1A (hpERF1A) suppressed ERF1A expression and ethylene biosynthesis by affecting *PpACO11* expression and PpSAMS1 protein abundance, ultimately altering ethylene signaling. The suppression of ERF1A enhances fruit softening by regulating the expression of *PpCLSE1*, *PpFUT1*, and *PpGDSL3*, and by decreasing the protein levels of PpGDSL3 and PpPME3. ERF1A silencing affects color formation by altering the biosynthesis of flavonols (e.g. quercetin) and anthocyanins (e.g. delphinidin), as well as the expression of flavonoid-related genes (*PpCYP450*, *PpHIDH*, *PpMYB4*, and *PpWRKY40*) and the protein levels of PpHIDH. Also, ERF1A modulates ABA and auxin levels by regulating the ABA biosynthesis gene *PpFMO*, the protein PpASR2, along with auxin biosynthesis genes *PpPIP2* and *PpCEP14*. ERF1A silencing increases SA content by reducing *PpSABP2* and *PpAATP* expression and protein abundances of PpSABP2, PpSABP2.1, and PpSABP2.2. Reduced ERF1A expression influences *PpCXE11* and *PpCXE13* transcription levels as well as PpCXE13 protein abundance, affecting ester (4-hexen-1-ol acetate (z)) and aldehyde levels (2-hexenal, heptanal, 2-heptenal, and octanal) to alter volatile balance. Abbreviations: ERF1A, ethylene response factor 1; *PpCLSE1*, cellulose synthase-like protein E1; *PpFUT1*, galactoside 2-alpha-L-fucosyltransferase isoform X1; *PpGDSL.3*, GDSL-like Lipase/Acylhydrolase superfamily protein; PpPME3, pectin methylesterase 3; *PpACO11*, 1-aminocyclopropane-1-carboxylate oxidase homolog 11; PpSAMS.1, *S*-adenosylmethionine synthase; *PpCYP450*, cytochrome P450; *PpHIDH*, 2-hydroxyisoflavanone dehydratase; *PpMYB4*, myb-related protein Myb4; *PpWRKY40*, probable WRKY TF 40; *PpCXE11*, carboxylesterase 11; *PpCXE13*, carboxylesterase 13; *PpFMO*, flavin containing monooxygenase; PpASR2, abscisic stress-ripening protein 2; *PpPIP2*, PAMP-induced secreted peptide 2; *PpCEP14*, C-terminally encoded peptide; *PpSABP2*, salicylic acid-binding protein 2; *PpAATP*, AAA-ATPase; ABA, abscisic acid; SA, salicylic acid.

Based on the ERF1A binding sites in the promoter region, 22 genes were selected as putative ERF1A target genes (Supplementary Table S8). The expression of these target genes during ripening was examined in ERF1A-silenced fruit (Fig. 7A). Data indicated that the expression of putative target genes was influenced by ERF1A silencing in a time-dependent manner (Fig. 7A), leading us to assume that most of the selected genes may be potential ERF1A targets. Motivated by the ripening changes observed in UV-C-treated peach fruit following ERF1A silencing (Fig. 5A), we also identified candidate genes associated with fruit firmness (cellulose synthase-like protein E1, *PpCLSE1*; galactoside 2-alpha-L-fucosyltransferase isoform X1, *PpFUT1*; *PpPME3*; and GDSL-like lipase/acylhydrolase superfamily protein, *PpGDSL3*), ethylene biosynthesis/signaling (ethylene-responsive TF 1B, *PpERF1B* and *PpACO11*), and color development (MYB-related protein MYB4, *PpMYB4* and probable WRKY TF 40, *PpWRKY40*). Based on the metabolic changes observed in the silenced fruit (Fig. 5B), we also analyzed the expression of *PpAMY*, 2-hydroxyisoflavanone dehydratase (*PpHIDH*), cytochrome P450 (*PpCYP450*), UDP-glycosyltransferase 76F1 (*PpUGT76F1*), and 2-alkenal reductase (NADP(+)-dependent) (*PpP2*), due to their established roles in ripening-related metabolic pathways (Fig. 3C; Shimamura et al. 2007; Wu et al. 2020; Zhang et al. 2021). We found that the expressions of *PpCLSE1*, *PpFUT1*, *PpGDSL3*, *PpACO11*, *PpMYB4*, *PpWRKY40*, *PpHIDH*, and *PpCYP450* were lower in ERF1A-silenced fruit 4 d following UV-C (Fig. 7A; Fig. 8).

Considering the high number of ERF1A putative target genes related to hormones (viz. *PpCRK*, *PpFMO, PpZRK, PpPIP2, PpCEP14, PpAATP*, and *PpSABP2*) (Fig. 7A; Fig. 8), we further explore the role of ERF1A in hormone homeostasis. Levels of auxin and salicylic acid (SA) increased, while ABA appeared to decrease in ERF1A-silenced peels (Fig. 7B). Because the ERF1A silencing affected several proteins related to fatty acid biosynthesis (i.e. long chain acyl-CoA synthetase 1, PpLACS1 and biotin carboxyl carrier protein of acetyl-CoA carboxylase 2, PpBCCP2) (Fig. 6C) and also triggers both *PpCXE11* and *PpCXE13* expression as well as PpCXE13 abundance (Fig. 6D; Fig. 7A), and considering the important role of these genes/proteins in volatile compound biosynthesis (Cao et al. 2019), we analyzed the peel-derived volatiles (Supplementary Fig. S7; Supplementary Table S9). ERF1A-silenced fruit accumulated greater amounts of various volatiles, including 2-hexenal, heptanal, and 2-heptenal, whereas other volatile compounds such as benzaldehyde, decanal, and ethylbenzene were less abundant (Fig. 7C), proposing the involvement of ERF1A in their regulation.

To determine whether some of the putative target genes are transcriptionally regulated by ERF1A, we performed a GUS reporter assay on *PpCXE11*, *PpCXE13*, and *PpSABP2*. The results revealed that all 3 genes exhibited increased fluorescence when co-introduced into protoplasts with the ERF1A overexpression plasmid (Fig. 7D), suggesting that ERF1A enhances the promoter activity of these genes. The transformation efficiency of the protoplasts was verified by the fluorescent microscopy using a GFP-containing vector (Fig. 7E), while the accumulation of ERF1A protein was confirmed through Western blot analysis (Fig. 7F).

## Discussion

The use of UV-C radiation as a ripening regulator has emerged as a key area of interest (Zhao et al. 2017; Michailidis et al. 2019; Zhang and Jiang 2019; Kan et al. 2021); however, the underlying mechanisms through which UV-C influences fruit biology remain unclear. As a starting point for our study, we demonstrated that UV-C influences key traits associated with peach ripening. Particularly, UV-C may temporarily induce respiration and ethylene production (Fig. 1A), accompanied by a rise in the expression of ethylene-associated genes, such as *PpACS.1* and *PpACO11* (Fig. 2C). This observation supports previous reports (Severo et al. 2015; Chen et al. 2022) indicating that UV-C enhances ethylene accumulation and induces ethylene biosynthetic gene expression in various climacteric fruits. The analysis through post-cold fruit ripening indicates that fruit softening, a major ripening-associated phenomenon in peaches (Tanou et al. 2017), was reduced by UV-C treatment (Fig. 1B), consistent with previous findings (Kan et al. 2021). Immunolocalization using anti-AGP antibodies revealed that UV-C increased AGPs, as indicated by JIM13, LM14, and LM30 distribution. Given the adhesive role of AGPs in the cell-wall structure during fruit softening (Leszczuk et al. 2020), the observed increase in AGPs in UV-C-treated fruit, along with their altered firmness, supports a role for AGPs in the delayed softening of peach fruit. Additionally, UV-C stimulated the presence of xyloglucan-specific epitopes recognized by LM25 antibody (XLLG, XXLG, and XXXG oligosaccharides of xyloglucan) and upregulated xyloglucan-related genes, including xyloglucan galactosyltransferase GT14 (*PpGT14.1*, *PpGT14.2*, and *PpGT14.3*) (Fig. 1C; Fig. 2B). Moreover, UV-C reduced unmethylesterified HGs, as detected by the LM19 antibody (Fig. 1C). During peach fruit softening, the action of pectin methylesterases reduces pectin methylation, facilitating further cleavage by pectic and pectate lyases, as well as polygalacturonases (Lurie et al. 2003). However, the reduced demethylesterification observed in UV-C-treated fruit suggests a suppression in softening due to the limited accessibility of these enzymes. Overall, our findings support the role of UV-C in cell-wall reprogramming, as demonstrated by the broad modulation of cell-wall-related genes in UV-C-treated fruit (Fig. 2B).

While comprehensive transcriptome investigation of UV-C in fruit ripening is limited (Zhao et al. 2017), our study identifies genes regulated by UV-C within a few hours after application, providing insights into the initial molecular events that might be triggered by UV-C. A key aspect of our study was the separate sampling of peel and flesh to reveal UV-C-induced changes driving the ripening transition in each tissue. The highest number of unique DEGs was found in the peel (Fig. 2A), suggesting that UV-C mainly affects the outer layer of the peel rather than the mesocarp cells of the peaches. This is probably linked to the presence of UV filtering compounds, blocking the in-depth penetration of UV-C irradiation (Guerrero-Beltrán et al. 2004). Hence, a challenging question is how UV-C regulates many genes in the flesh (Fig. 2A) and postpones the flesh-softening process (Fig. 1B). Peach fruit peel is empowered with a sensory mechanism to counteract various environmental stressors (Karagiannis et al. 2016). Therefore, it is probable that high-energy UV-C levels may be sensed by the peel and then transformed into organized local and systemic responses, leading to the whole fruit's metabolism reset. Consequently, UV-C reception in peel not only activated gene expression but also induced systemic suppressive effects in the flesh, which resulted in an extended biologically relevant ripening effect, as indicated by the softening inhibition that lasted for at least 9 d (Fig. 1B).

Our data also support the establishment of a UV-C-associated reprogramming of central and secondary metabolism. Fruit treated with UV-C displayed alterations in the expression of genes involved in polyphenol biosynthesis that were accompanied by higher levels of anthocyanin, such as delphinidin in the peel (Supplementary Fig. S3B), consistent with the changes in peel color indicators and fruit phenotype (Fig. 1A). These findings altogether could be linked to UV-C-accelerated fruit coloring (Zhao et al. 2017). Data also revealed an accumulation of several sugars, such as sucrose and maltose, in both UV-C-treated tissues (Supplementary Fig. S3A) and the activation of their biosynthetic genes (Fig. 3C), confirming previous findings (Michailidis et al. 2019). Genes involved in abscisic acid (ABA), jasmonic acid, and brassinosteroid metabolism were affected by UV-C treatment (Fig. 3A), indicating a regulation in phytohormone signaling.

Emerging evidence indicates that fruit ripening is epigenetically regulated, with gene expression closely linked to DNA methylation status (Ji and Wang 2023). Consistent with previous studies (Willing et al. 2016; Xiang et al. 2017), our data show that UV-C induces both DNA N6-methyldeoxyadenosine (6mA) and RNA m6A methylation (Fig. 4B). While further research is needed to fully characterize the epigenetic (DNA) and epitranscriptomic (RNA) impacts of UV-C in peach ripening, our findings suggest that it may reprogram gene expression by promoting adenine methylation, potentially reshaping ripening signals. Given the UV-C-stimulated DNA methylation observed during peach ripening (Fig. 4B), the known sensitivity of ERFs to methylation during fruit ripening (Han et al. 2016) and reports of UV-C-induced mutations (Sun et al. 2010; Nakamura et al. 2021), we investigated whether ERF1A undergoes targeted methylation and/or mutation to clarify its role in UV-C-mediated ripening. Bisulfite sequencing showed no UV-C-inducible cytosine methylation in ERF1A, and coding region analysis confirmed the absence of UV-C-induced mutations (Fig. 4C). Overall, our findings show that ERF1A function remains largely unaffected by UV-C at the epigenetic or genetic level, supporting its use in further studies.

This study shows that UV-C induces transcriptomic reprogramming in peach fruit (Fig. 2), delaying ripening (Fig. 1) alongside the upregulation of ripening-related TFs, notably *ERF1A* (Fig. 2D). Other UV-C-responsive TFs, *PpERF2* and *PpERF4.1* (Figure 2D), clustered closely with ERF1A in the phylogenetic tree (Fig. 4D). Early studies in tomatoes show that ERF regulates fruit ripening (Liu et al. 2016), while UV-C modifies the expression of numerous ERFs (Severo et al. 2015; Chen et al. 2022). Studies of ERF genes and their expression profiles have been reported in peach fruit (Zhou et al. 2020), although there is a lack of functional assessment. Given also the recent findings linking ERF1A to ethylene production and anthocyanin biosynthesis during climacteric fruit ripening (Li et al. 2023), we selected it as a primary target to investigate the role of UV-C in peach ripening. Thus, we transiently knocked down ERF1A expression through RNAi and analyzed its effects on peach ripening following UV-C exposure. Silencing *ERF1A* led to its downregulation (Fig. 5A), suppressed ethylene biosynthesis (Fig. 5A), and reduced PpSAMS.1 protein accumulation (Fig. 6C), suggesting ERF1A as a key regulator of the UV-C ripening response and its link to ethylene signaling. We further present several lines of evidence substantiating that the ERF1A silencing before UV-C exposure reversed a large part of the UV-C-delayed ripening responses. Fruit softening in ERF1A-silenced (hpERF1A) peaches was significantly greater than in GFP controls (Fig. 5A). This enhanced softening aligns with the upregulation of multiple cell-wall-degrading enzymes (Fig. 6C), which drive the observed changes in pectin, xyloglucan, and AGPs (JIM13, LM14, and LM30) composition (Fig. 5C). In particular, hpERF1A fruit accumulated higher levels of demethylesterified HGs (LM19 signal), indicating that pectin de-esterification and subsequent cleavage proceed unimpeded in these samples. Demethylesterified HGs are inherently more susceptible to attack by polygalacturonases and pectate lyases, thereby facilitating the solubilization phase of ripening (Wang et al. 2018a). Finally, the detection of LM25 epitopes confirms that xyloglucan breakdown and pectin solubilization occur in their characteristic sequential order, first xyloglucan loosening, then pectin dissolution (Muramatsu et al. 2004), demonstrating that ERF1A silencing does not block the normal progression of cell-wall remodeling during softening. Meanwhile, the loss of firmness, despite the decrease in ethylene production in silenced fruit (Fig. 5A), denotes that ERF1A could regulate softening through ethylene-independent pathways, possibly through the activation of specific genes and proteins (Fig. 8). The reversed effect of ERF1A in UV-C ripening outcome was further illustrated by the increase in water loss (Fig. 5A), which was accompanied by changes in aquaporin (PpPIP2-2) and desiccation-related protein (PpPCC13-62) in silenced fruit (Fig. 6C; Fig. 6D). ERF1A-silenced fruit displayed greater proportional weight loss, possibly due to the change in fruit cell size (Fig. 5A), which may be linked to the softening of silenced fruit, as previously suggested in peach fruit (Ghiani et al. 2011). Impairing the expression of ERF1A was also effective in reversing the effect of UV-C in the peel central metabolism. In contrast with the accumulation of sugars observed above in UV-C-exposed fruit (Supplementary Fig. S3C), lower concentrations of most sugars, such as maltose, sucrose, and trehalose, were recorded in ERF1A-RNAi peels (Fig. 5B). Current data also indicated that silenced fruit had faint pigmentation (Fig. 5A), as demonstrated by the reduction of color indicators, consistent with the lower levels of quercetin (a yellow-colored pigment flavonol) and delphinidin (a purple-colored anthocyanin pigment) (Fig. 5B; Fig. 8). These data provide evidence that the UV-C ripening outcome was disrupted by transient ERF1A silencing and support the pleiotropic effect of ERF1A on peach ripening, as well as its potential role in other *Prunus* species with homologous ERF1A-like sequences (Fig. 4E).

Another major target of interest in this study was the alteration of the peel proteomic profile in response to ERF1A silencing. It is worth emphasizing not only the relatively high number of proteins whose accumulation altered in ERF1A-RNAi fruit (Fig. 6A) but also the functional categories to which they can be assigned, with most of the overaccumulated proteins being associated with oxidative stress and ROS metabolism (Fig. 6B), including various heat shock proteins, such as PpHSP18.1A, PpHSP18.5.2, and PpHSP17.8 (Figure 6C). These findings indicate an earlier onset of ripening in ERF1A-silenced fruit and are consistent with the generation of oxidative stress conditions (Fig. 6B). A meta-analysis comparison between the RNA-seq data of UV-C-exposed peel (Fig. 2A) with the proteomic dataset of UV-C-treated ERF1A-silenced samples (Fig. 6A) identified 41 UV-C-responsive genes whose corresponding proteins were also affected in ERF1A-silenced peel tissue (Fig. 6D) and therefore could be considered putative ERF1A targets. Interestingly, ERF1A silencing induced strong differences between mRNA expression and their protein quantities (85.4%; 35 gene-to-protein pairs exhibited different trends), suggesting that the UV-C-responsive transcriptome and proteome can be shaped differently depending on ERF1A expression levels. It should be noted that, although these 2 datasets were derived from different experimental conditions, and the association between mRNA abundance and protein levels may be influenced by posttranscriptional regulation, their integration proved highly informative for identifying candidate *ERF1A* targets. Another notable finding from this proteogenomic analysis was the increase of abscisic stress-ripening protein 2 (PpASR2) in silenced fruit, while the corresponding genes were downregulated in UV-C-exposed fruit (Fig. 6D). Also, various SA-binding proteins, such as PpSABP2 and PpSABP2.1, were underaccumulated in RNAi-ERF1A fruit, while UV-C upregulated the expression of their genes. Consistent with this, enriched groups of genes related to hormone signaling, including response to ABA, were affected by UV-C (Fig. 3A). This regulation pattern raises the hypothesis that a fundamental shift in hormone signaling and/or interplay occurs in ERF1A-silenced fruit.

Another insight brought by our study is related to the characterization of the putative targets of ERF1A in peach fruit. Using information about the genomic positions of the ERF1A binding sites, we identified 22 genes as potential targets of this TF. RT-qPCR analysis in ERF1A-silenced peel revealed that a great number of these genes were downregulated for at least one of the ripening stages examined (Fig. 7A), proposing that these genes might serve as possible targets of ERF1A. In addition to identifying potential *ERF1A* target genes based on binding sites, we examined a group of ripening-related genes involved in fruit firmness, color development, and ethylene production. Silencing ERF1A reduced the expression of firmness-related genes *PpCSLE1* (Hu et al. 2025), *PpFUT1* (Wang et al. 2018b), and *PpGDSL.3* (Pei et al. 2019) (Fig. 7A; Fig. 8), supporting its role in delaying ripening. *PpACO11* expression also declined in silenced fruit (Fig. 7A; Fig. 8), consistent with reduced ethylene production (Fig. 5A) and previous findings (Li et al. 2023). Additionally, color differences between silenced and control fruit (Fig. 5A) likely result from downregulation of *PpMYB4* and *PpWRKY40* (Fig. 7A; Fig. 8), both associated with anthocyanin accumulation (An et al. 2019). Our study also shows that impairment of ERF1A results in downregulation of the *PpHIDH* and *PpCYP450* (Fig. 7A; Fig. 8), known to control the isoflavonoid biosynthesis (Shimamura et al. 2007), further supporting the observed link between ERF1A and secondary metabolism (Fig. 5B).

An interesting outcome in this study was the observation that the ERF1A candidate target genes whose expression was decreased by silencing (Fig. 7A, Fig. 8) are involved in hormone signaling, including ABA (*PpFMO*, Wang et al. 2023) and auxin (*PpPIP2*, Hussain et al. 2021; *PpCEP14*, Roberts et al. 2013). This observation is aligned with the role of auxin and ABA in AP2/ERF ripening function (Xie et al. 2019) and peach ripening (Wang et al. 2019; Ma et al. 2023). Further evidence for the impact of ERF1A in hormone reconfigurations arose from the observation that auxin levels were increased, while ABA was decreased in the silenced peel (Fig. 7B; Fig. 8). These results are consistent with a pattern in which peach fruit ripening (Fig. 5A) is associated with changes in auxin and ABA levels (Wang et al. 2019; Ma et al. 2023). *PpERF3* has been shown to function as a transcriptional activator by binding to ERF response elements in the promoters of ABA biosynthesis genes in peach fruit during ripening, thereby increasing ABA levels (Wang et al. 2019). Although our peel-based hormone data do not allow us to draw a general conclusion about the ripening process of the whole fruit, they clearly suggest that impairing ERF1A expression results in hormone reprogramming, which could explain the distinct ripening behavior of the silenced fruit (Fig. 5A).

There is evidence, although still not extensive, that several *ERF* genes are SA inducible (Caarls et al. 2017). Herein, we provide evidence for a link between the repressor ERF1A and SA during peach ripening. First, several presently identified as putative ERF1A targets, including *PpAATP* and *PpCRK* (Fig. 7A; Fig. 8), have been recognized as key components of SA signaling (Segarra et al. 2010). Second, our data show that the salicylic acid-binding protein 2 (*PpSABP2*), a receptor for SA, was decreased in ERF1A-RNAi fruit at all conditions assessed (Fig. 7A; Fig. 8). In support, GUS experiments indicated that ERF1A acts as a transcriptional activator of *PpSABP2* by enhancing its promoter activity (Fig. 7D). Additionally, we detected a strong reduction of PpSABP2, PpSABP2.1, and PpSABP2.2 proteins in ERF1A silencing fruit, confirming that not only this gene but also the gene products are affected by ERF1A (Fig. 6D; Fig. 8). To this end, SA content was induced in silenced fruit (Fig. 7B; Fig. 8). These findings suggest that ERF1A regulates SA signaling via both transcriptional and posttranscriptional activation of *PpSABP2* (Fig. 8).

Carboxylesterases (CXEs) catalyze the hydrolysis of a carboxylic ester to alcohol and carboxylic acid anion. Previous studies in peach fruit (Cao et al. 2019) reported that CXEs are associated with the catabolism of volatile esters, such as hexyl acetate, *E*-hexenyl acetate, and *Z*-3-hexenyl acetate. Our study uncovers a link between ERF1A and CXEs during peach ripening. Indeed, a strong downregulation of *PpCXE11* and *PpCXE13* expression, in conjunction with underaccumulation of PpCXE13 protein in ERF1A-silenced peaches, indicates that *PpCXE11* and *PpCXE13* represent putative targets of ERF1A (Fig. 6D; Fig. 7A; Fig. 8). This was further validated by the GUS assay, showing that ERF1A positively regulates the promoting activity of the 2 CXEs (Fig. 7D). Our study also shows that ERF1A silencing induced proteins related to fatty acid biosynthesis, such as PpLACS1 and PpBCCP2 (Fig. 6C), suggesting that ERF1A may modulate fatty acid metabolism to produce volatile compounds (Fig. 8). Our chromatographic analysis of volatiles revealed that ERF1A suppression decreased 4-hexen-1-ol acetate (z) ester and increased aldehydes/alcohols, such as 2-hexenal, heptanal, 2-heptenal, and octanal (Fig. 7C), proposing that the decreased levels of CXEs in ERF1A-RNAi fruit provoke alcohol accumulation by hydrolyzing esters (Fig. 8). Although we cannot exclude the possibility that some of these volatiles may be affected by the accelerated ripening of silenced fruit (Fig. 5A), the above evidence highlights the contribution of ERF1A to volatile regulation in peach fruit (Fig. 8).

## Conclusions

This study reveals the tissue-specific factors linking UV-C treatment to ripening delay in peach fruit. Our findings demonstrate that ERF1A is responsive to UV-C and contributes to ripening inhibition by regulating target genes (*PpCXE11*, *PpCXE13*, and *PpSABP2*), protein abundance, metabolite production (sugars, phenolics, and volatiles) and key ripening traits such as softening, coloration, and hormone metabolism, particularly involving indole-3-acetic acid (IAA), ABA, SA, and ethylene signaling. Building on these results, we propose a model illustrating the interaction between UV-C and ERF1A in peach fruit ripening (Fig. 8). Overall, our study identifies key molecular components and mechanistic pathways through which UV-C and ERF1A modulate the ripening transition in peach fruit. However, further research is required to clarify the regulatory role of ERF1A under physiological (non-UV-C) conditions. In addition, it remains to be determined whether the UV- and ERF1A-mediated regulatory network is conserved across other climacteric fruit species. These findings contribute to a deeper understanding of ripening control and support targeted breeding strategies aimed at improving fruit quality.

## Materials and methods

### Fruit material and UV-C treatment

Nectarine (*P. persica* L. Batsch, cv. Luciana) fruit was harvested from a commercial orchard in North Greece (Velventos, Kozani region). Uniform fruits were immediately transferred to the Pomology Laboratory of Aristotle University of Thessaloniki and randomly divided into 2 groups of 150 fruits each. The first group was exposed to UV-C irradiation for 10 min (5 min—6 kJ m^−2^ on each side) in a modified machine with 4 UV-C lamps attached (OSRAM HNS 55W OFR G13, 26 mm diameter, and 895 mm length) while the second group remained untreated (control). The selection of the parameters regarding the UV-C application was based on preliminary experiments and our previous work (Michailidis et al. 2019). Each treatment/group (UV-C and control) was divided into 2 subgroups, of which the first subgroup remained at room temperature (20 °C) for 8 d (herein defined as “ripening”), while the second subgroup was cold stored (0 °C, 95% RH) for 7 d and then allowed to ripen at room temperature (20 °C) for 2 d (defined as “post-cold ripening”). Fruit ripening characterization was conducted at 1, 2, 4, 6, and 8 d of ripening (20 °C) for the first subgroup, and for the second subgroup (i) 8 h after UV-C application (8 h, before cold storage), (ii) after cold storage (0 °C) following the stabilization of fruit temperature (0 d), as well as (iii) after 2 d of post-cold ripening (20 °C, 2 d). Samples of the mesocarp (flesh) and exocarp (peel) were collected, frozen with liquid nitrogen, and stored (−80 °C). A schematic illustration of the overall experimental design is provided in Supplementary Fig. S8.

### Evaluation of fruit ripening progress

Fruit weight loss (%) (18 fruits for each subgroup) was determined during various ripening times and conditions as the decrease in fruit weight compared to harvest. Peel color indicators L*, a*, and b* were measured using a colorimeter (Minolta CR-200 Minolta, Japan), and the indicators *Chroma* (C*) and *Hue angle* (*H°*) were calculated (Tanou et al. 2017). A texture analyzer (TA.XT plusC, Stable Micro Systems, UK) was used to determine fruit firmness (Polychroniadou et al. 2022). Respiration and ethylene production were analyzed by a gas chromatographer (Shimadzu GC-2014, Kyoto, Japan), as described in Minas et al. (2018). Soluble solids concentration (SSC, %) and titratable acidity (% malic acid) were determined (Tanou et al. 2017). All ripening analyses were performed using 9 fruits per treatment (3 fruits × 3 replicates).

### Electrical conductivity estimation

Electrical conductivity was measured as described by Polychroniadou et al. (2022) using 9 fruits (3 fruits × 3 replicates) for each treatment.

### Cell wall epitope immunolocalization and specimen visualization

Semi-thin sections (0.5 to 2 *μ*m thick) of fruit were prepared according to Meidani et al. (2019). Sections were mounted onto glass slides and subjected to either staining with 0.5% (w/v) toluidine blue or immunostaining for pectin and AGPs detection. The localization of pectins was achieved using the LM19 primary antibody, a rat IgM monoclonal antibody that specifically binds to pectic epitopes, and the LM20 antibody (Supplementary Table S1). For AGP detection, 3 primary antibodies were employed: JIM13, which is a rat IgM monoclonal antibody binding to the β-GlcA-(1 → 3)-α-GalA-(1 → 2)-α-Rha motif of AGPs and recognizing the AGP2 epitope found in various plant exudates (PlantProbes, University of Leeds, UK; Supplementary Table S1), LM14, which is an antibody recognizing arabinose- and galactose-enriched carbohydrate chains present in AGPs or rhamnogalacturonans (RGs-I) side chains (Moller et al. 2008); and LM30, another rat IgM monoclonal antibody that targets AGP epitopes (Wilkinson et al. 2017; Supplementary Table S1). Xyloglucan-specific epitopes were recognized by the LM25 antibody (XLLG, XXLG, and XXXG oligosaccharides of xyloglucan; Pedersen et al. 2012). The immunolocalization procedure was performed as described by Adamakis et al. (2014). To detect cellulose, a subset of sections was also stained with Calcofluor-white (Calco). Microscopy and image acquisition were performed using a Zeiss Axioplan microscope with LSM780 confocal laser scanning microscopy capabilities (Carl Zeiss AG). FITC was excited at 488 nm, and emission was collected using a 525/50 nm bandpass filter, with laser power and detector gain adjusted to avoid saturation while maintaining signals within the linear range. Calcofluor White was excited using a 405 nm laser (UV 355 nm where available), and emission was collected with a 450/50 nm bandpass filter under reduced laser power due to the brightness of the dye. Single-stained controls were used to optimize photomultiplier tube voltages and to confirm minimal spectral overlap between the 2 fluorophores (Pappas et al. 2022). Fluorescence intensity was calculated according to Adamakis et al. (2014). For each treatment, fluorescence intensity was measured in 5 sections from 3 different fruits, and the mean values were calculated accordingly.

### Transcriptome analysis by RNA-seq

Total RNA was extracted using Monarch Total RNA Miniprep Kit (New England BioLabs Inc., Ipswich, MA, USA) (Polychroniadou et al. 2022) from flesh and peel tissue, which were sampled in triplicate (3 fruits per biological replicate) at 8 h following UV-C treatment. Poly-A-enriched libraries were prepared from the isolated RNA using the Illumina TruSeq Stranded RNA Library Prep Kit. Quantification and qualification of the sample library were performed using an Agilent 5300 Fragment Analyzer. An Illumina Novaseq 6000 platform was used for library sequencing and Trim Galore for trimming sequences for adaptors and filtering low-quality reads and unknown sequences. *P. persica* Zhongyoutao 14 Genome v1.0 (Lian et al. 2022) was used as the reference genome for paired-end reads alignment. HTSeq v0.11.1 was used for the quantification of the raw read counts using the “-s reverse–type=gene” option. “Transcripts Per Million (TPM)” was used to normalize raw data. The normalized data were filtered to leave only the always-above-0 genes, and then it was log_2_ transformed and scaled using *z*-score. Differential expressions between samples were determined as described (Robinson et al. 2009) using the edgeR. DEGs were considered those with a *q*-value < 0.01. RT-qPCR was performed to support RNA-seq expression dynamics (Supplementary Fig. S2).

### Gene expression analysis by RT-qPCR

Monarch Total RNA Miniprep Kit (New England Biolabs) was used to extract total RNA from samples 8 h after UV-C treatment. LunaScript RT SuperMix Kit (New England Biolabs) was used for cDNA synthesis through reverse RNA transcription in a PCR system (ProFlex, Thermo Fisher Scientific). The analysis was performed in a QuantStudio 5 Real-Time PCR System (Thermo Fisher Scientific), as described in Polychroniadou et al. (2022).

### Analysis of primary and secondary metabolites

The analysis of primary metabolites was conducted using a GC-MS (PerkinElmer Clarus SQ 8S) in peel and flesh samples at 4 and 8 d of ripening and at 0 and 2 d of post-cold ripening after UV-C treatment. The extraction procedure, GC/MS program, and analysis were described in detail by Skodra et al. (2023). Results were expressed as a relative abundance of adonitol. Secondary metabolite analysis was performed using Agilent 1260 Infinity II (Agilent Technologies, Santa Clara, CA, USA). Phenol extraction, LC-MS program, and analysis were performed according to Irakli et al. (2021), while the procedures for anthocyanins were described by Kalogiouri et al. (2022). OpenLab LC-MS software (Agilent Technologies) was used for data acquisition and processing, while standard compounds were used for identification. Three biological repeats were conducted; each one consisted of 3 fruits per treatment.

### DNA 6mA and RNA m6A **assays**

Genomic DNA (100 ng) was extracted from peel tissue at 8 h after UV-C treatment as well as at 0 d of post-cold ripening. To quantify DNA 5mC and m6A RNA methylation, the MethylFlash Global DNA Methylation ELISA Easy Kit and the EpiQuik m6A RNA Methylation Quantification kit were used, respectively, according to the manufacturer's instructions, using a Varioskan LUX microplate reader.

### Targeted bisulfite sequencing experiment of ERF1A

Genomic DNA was extracted from peel tissue at 1 d following UV-C treatment according to Dalakouras et al. (2016). Approximately 500 ng was used for bisulfite treatment using the EZ DNA Methylation-Gold Kit according to the manufacturer's instructions and as described before (Dalakouras et al. 2016) using the primers 5′-ATG TTY GGA YAG AGT GGA AYA GA-3′ and 5′-TCC TCC TTR ARC RRC AAA TCR CCC C-3′. The amplicons were cloned into pGEM-T Easy and sequenced. Sequencing data were analyzed with Cymate software (Hetzl et al. 2007).

### Phylogenetic analysis of *P. persica* ERFs and ERF1A among *Prunus* species

The evolutionary relationship among the members of the *P. persica* AP2/ERF superfamily was studied. The genes were selected based on the analysis of Zhang et al. (2012) and by the identification of the AP2 domain in the CDS of other *P. persica* genes through the NCBI Conserved Domain Database. The CDS of ERF1A (1287 bp—Sanger sequencing) were retrieved through the Genome Database for Rosaceae (GDR) (Supplementary Table S6). Moreover, the evolutionary relationship of ERF1A among different *Prunus* species was studied. The genome of each *Prunus* species and the CDS of ERF1A were also retrieved through GDR (Supplementary Table S6). The analysis and design of the phylogenetic trees were performed using the software MEGA (v12) by selecting the model Maximum Likelihood. The trees are displayed as cladograms, with branch lengths normalized for visualization; the figures are intended to illustrate the topology of sequence relationships.

### Vector construction and RNAi silencing of ERF1A in peach fruit

For the generation of pCambia-hpERF1A (Supplementary Fig. S9), a 269 bp ERF1A CDS fragment (specifically designed on *Pp05G018230* sequence) was inserted into the pCambia1300 vector (Addgene, www.addgene.org) in sense and antisense orientation. Agroinfiltration of nectarine fruit cv. Morsiani 90 was performed using a vacuum pump (Electric Aspirator VE-11, Jeio Tech) set to −90 kPa. For control treatment, a pCambia 1300 vector carrying a GFP hairpin construct was used (Supplementary Fig. S9; Dalakouras et al. 2023). Three days after RNAi application, all infiltrated fruit (hpERF1A, hpGFP) were subjected to UV-C treatment, as described above, and then allowed to ripen at room temperature for 4 d (Supplementary Fig. S8). Fruit ripening characterization and sampling of ERF1A-silenced peel were conducted at 1, 2, 3, and 4 d after UV-C treatment C (4, 5, 6, and 7 d following ERF1A silencing, respectively). To validate the silencing efficiency, we assessed the expression levels of *ERF1A* by RT-qPCR in ERF1A-silenced peel tissue, compared to the peel tissue of control fruits (hpGFP), using primers located outside the RNAi target region (ERF-off), at the same time points used for ripening characterization. RNAi metabolomic analysis was performed for the peel tissue of the latter time point, as described previously.

### Protein identification in ERF1A-silenced fruit

Proteins of ERF1A-silenced peel at 1 and 4 d after UV-C treatment were extracted and processed for proteomic analysis according to the sp3 tryptic digestion protocol (Skodra et al. 2023). Samples were run on a liquid chromatography tandem mass spectrometry (LC-MS/MS) setup consisting of a Dionex UltimateRSLC online with a Thermo Q Exactive HF-X Orbitrap mass spectrometer (Thermo Scientific) (Bekas et al. 2023). Orbitrap raw data were analyzed in DIA-NN v1.8.1 (Data-Independent Acquisition by Neural Networks) (Demichev et al. 2020) through searching against the *P. persica* CN14 protein database containing 35,355 proteins (Lian et al. 2022) using the library-free mode of the software and allowing up to 2 tryptic missed cleavages. A spectral library was created by the DIA runs and used to reanalyze them. DIA-NN default settings have been used with oxidation of methionine residues and acetylation of the protein N-termini set as variable modifications and carbamidomethylation of cysteine residues as a fixed modification. N-terminal methionine excision was also enabled. The match between runs feature was used for all analyses, and the output (precursor) was filtered at 0.01 FDR. Finally, the protein inference was performed on the level of genes using only proteotypic peptides. Results were processed statistically and visualized by Perseus software (1.6.15.0).

### Analysis of ERF1A target genes in ERF1A-silenced fruit

The identification of genes targeted by ERF1A was determined using the Plant Promoter Analysis Network v3.0 (Chang et al. 2008). Briefly, the accession number of ERF1A and the binding site “GCCGC” were used to search cis-regulatory elements in the promoter using the *Arabidopsis thaliana* weight matrix. The expression of ERF1A and its candidate target genes was assayed in peel samples by RT-qPCR at 1, 2, 3, and 4 d of ripening following UV-C treatment, after ERF1A silencing.

### IAA, ABA, and SA determination

Estimation of IAA, ABA, and SA content was performed at 4 d of ripening after UV-C treatment following ERF1A silencing, by using 0.2 g of freeze-dried skin tissue. Determination was conducted in a Tecan Infinite M200PRO microplate reader (Tecan, Switzerland) using the Plant IAA ELISA Kit (ELK 8753), Plant ABA ELISA Kit (ELK 8563), and Plant SA ELISA Kit (ELK 8702) (ELK Biotechnology), according to the instructions of the manufacturer.

### Volatile organic compound analysis

For volatile compound analysis, peach skin tissue sampled (3 fruits × 3 replicates) at 4 d of ripening after UV-C treatment, following ERF1A silencing) was extracted as previously reported by Zhang et al. (2011). The analysis was carried out using a Trace GC Ultra 299 equipped with an ISQ MS and a TriPlus RSH autosampler (Thermo Fisher Scientific Inc., Switzerland), coupled with In-Tube Extraction Dynamic Headspace (ITEX-DHS), following the method previously described by Zhang et al. (2011).

### GUS reporter assay and western blot analysis

Approximately 500 to 1,000 bp of the candidate ERF1A-targeted genes (Carboxylesterase 11; *PpCXE11*, carboxylesterase 13; *PpCXE13*, and SA binding protein 2; *PpSABP2*) promoter sequences were inserted into pRT-GUS vectors using the primers described in Supplementary Table S10 (GUS vectors). The coding sequence of ERF1A was inserted into a pRT vector containing an in-frame 3xHA-tag sequence under the control of CaMV35S promoter (OE_ERF1A vector). A pRT plasmid carrying the neomycin phosphotransferase gene was used as a mock control (Neo vector). Vector sequences are shown in Supplementary Fig. S9. Protoplasts of *A. thaliana* leaves were isolated and transformed using the polyethylene glycol)-mediated transformation. A total of 50,000 protoplasts were transformed with a 10 *μ*g of plasmid DNA per sample, consisting of either 1 *μ*g of OE_ERF1A vector, 1 *μ*g of the GUS vectors, and 8 *μ*g of Neo vector or 1 *μ*g of OE_ERF1A vector and 9 *μ*g of Neo vector. Transformed protoplasts were determined by fluorescence (El-shershaby et al. 2019).

Western blot analysis was conducted by transforming 100,000 protoplasts with 10 *μ*g of OE_ERF1A vector and 10 *μ*g of Neo vector or 20 *μ*g of Neo vector (control). Following overnight incubation, protoplasts were observed by microscopy (Nikon C2+ Confocal Microscope), while a GFP-containing vector was transformed into the protoplasts to test the transformation efficiency. Protoplast proteins were extracted (Löchli et al. 2023), and electrophoresed proteins were transferred by semi-dry blotting to PVDF membranes using eBlot L1 (L00686C, GenScript). Membranes were incubated with primary antibodies Anti-HA (4F6, ABT2040-50UL, Abbkine Scientific) and Anti-Actin (3T3, ABL1050-50UL, Abbkine Scientific), and peroxidase-conjugated second antibody (Abclonal, AS003). Signals were detected by Pro-light HRP chemiluminescence detection, and images were taken using the Tanon5200 Tanon.

### Statistical analysis

SPSS (v25.0., Chicago, USA) was used for the statistical analysis, and significant statistical differences were detected based on Student's *t*-test (*P* ≤ 0.05). Principal component analysis and GO enrichment analysis were also performed. Visualization of enrichment plots was performed using SRplot.

### Accession numbers

The sequence data generated in this study are available in the GenBank/EMBL databases under the accession numbers listed in Supplementary Table S3.

## Supplementary Material

kiaf409_Supplementary_Data

## Data Availability

The data underlying this article are available in the article and in its online Supplementary material. The transcriptome data from this article have been deposited in the NCBI Sequence Read Archive in the Bio-Project PRJNA1180494. The mass spectrometry proteomics data have been deposited to the ProteomeXchange Consortium via the PRIDE partner repository with the dataset identifier PXD060506 (Perez-Riverol et al. 2019).

## Supplementary data

The following materials are available in the online version of this article.

**Supplementary Table S1.** Primary monoclonal antibodies used for immunolocalization of the different cell wall epitopes.

**Supplementary Table S2.** Tissue-specific RNA-seq analysis after UV-C treatment.

**Supplementary Table S3.** Abbreviations and accession numbers for genes expressed in peach fruit tissues.

**Supplementary Table S4.** Primary metabolites in peach fruit tissues during ripening.

**Supplementary Table S5.** Secondary metabolites in peach fruit tissues during ripening.

**Supplementary Table S6.** Phylogenetic analysis among members of AP2/ERF superfamily. ERF1A phylogenetic analysis among different Prunus species.

**Supplementary Table S7.** Proteomic analysis of UV-C-treated peel tissue in response to *ERF1A* silencing.

**Supplementary Table S8.** ERF1A-targeted genes.

**Supplementary Table S9.** Analysis of peel volatile compounds in *ERF1A-*silenced fruit.

**Supplementary Table S10.** List of primers used.

**Supplementary Figure S1.** Ripening traits and electrolyte leakage of peach fruit tissues during ripening.

**Supplementary Figure S2.** Validation of RNA-seq data by RT-qPCR.

**Supplementary Figure S3.** Primary and secondary metabolites in peach fruit tissues.

**Supplementary Figure S4**. Ripening features of “Morsiani 90” fruit in the absence or presence of cold storage following UV-C treatment.

**Supplementary Figure S5.** Relative expression of ERF1A in “Morsiani 90” peel after UV-C exposure (3 fruits × 3 replicates).

**Supplementary Figure S6.** Color indicators between hpERF1A and control peaches, represented as fold change (log_2_).

**Supplementary Figure S7.** Comparison of volatile organic compounds between hpERF1A and control peaches, represented as fold change (log_2_).

**Supplementary Figure S8.** A schematic representation of the experimental procedure.

**Supplementary Figure S9.** Sequences of vectors used.

## Dive Curated Terms

The following phenotypic, genotypic, and functional terms are of significance to the work described in this paper:

AGT Gramene: AT2G13360
AGT Araport: AT2G13360

MYB4 Gramene: AT4G38620
MYB4 Araport: AT4G38620

fruit AmiGo: PO:0009001

ACS Gramene: AT5G36880
ACS Araport: AT5G36880

ERF1 Gramene: Acc25620
ERF1 Araport: Acc25620

AGP2 Gramene: GRMZM2G027955
AGP2 Araport: GRMZM2G027955

GDR Gramene: https://www.rosaceae.org/
GDR Araport: https://www.rosaceae.org/

NCBI Gramene: https://www.ncbi.nlm.nih.gov
NCBI Araport: https://www.ncbi.nlm.nih.gov

fruit weight Planteome: TO:0002746
