# Supplementary material for: The ethylene response factor ERF1A regulates UV-C-induced delayed ripening in peach fruit
Source: Plant Physiol. 2025 Sep 22;199(2):kiaf409. doi: 10.1093/plphys/kiaf409 (PMC12501976; doi:10.1093/plphys/kiaf409)
Supplement: kiaf409_Supplementary_Data [file kiaf409_supplementary_data.zip › Supplementary References.docx]

**References**

Knox JP, Linstead PJ, Cooper JPC, Roberts K. Developmentally regulated epitopes of cell surface arabinogalactan proteins and their relation to root tissue pattern formation. *The Plant Journal.* 1991:1(3):317-326. 10.1046/j.1365-313x.1991.t01-9-00999.x

Lisec J, Schauer N, Kopka J, Willmitzer L, Fernie AR. Gas chromatography mass spectrometry-based metabolite profiling in plants. *Nature Protocols*. 2006:1(1):387–396. 10.1038/nprot.2006.59

Verhertbruggen Y, Marcus SE, Haeger A, Ordaz-Ortiz JJ, Knox JP. An extended set of monoclonal antibodies to pectic homogalacturonan. *Carbohydrate Research*. 2009:344(14):1858-1862. 10.1016/j.carres.2008.11.010
